# High performance asymmetric supercapacitors based on Ti_3_C_2_T_*x*_ MXene and electrodeposited spinel NiCo_2_S_4_ nanostructures†

**DOI:** 10.1039/d2ra00991a

**Published:** 2022-04-07

**Authors:** Mansi Pathak, S. R. Polaki, Chandra Sekhar Rout

**Affiliations:** Centre for Nano and Material Science, Jain University Jain Global Campus, Jakkasandra, Ramanagaram Bangalore-562112 India r.chandrasekhar@jainuniversity.ac.in csrout@gmail.com; Surface and Nanoscience Division, Materials Science Group, Indira Gandhi Centre for Atomic Research-Homi Bhabha National Institute Kalpakkam Tamil Nadu 603102 India

## Abstract

Spinel metal sulfides have been investigated for a wide range of applications mostly in electrochemical energy storage owing to their better electronic conductivity and high reversible redox activity. Herein, we report a facile fabrication approach for the binder-free supercapacitor electrodes based on spinel NiCo_2_S_4_ (NCS) on various substrates such as Cu-foil (CF), Ni-foam (NF), and vertical graphene nanosheets grown on carbon tape (VG) *via* a single step-controlled electrodeposition technique. The obtained electrodeposited NiCo_2_S_4_ grown on Cu-foil (denoted as CF-NCS) in symmetric assembly shows a high specific capacitance of 167.28 F g^−1^ compared to NCS grown on Ni-foam and VG substrates, whereas, symmetric NiCo_2_S_4_ grown on a VG substrate device exhibits better cycling performance (81% for 3000 cycles) compared to CF-NCS and NF-NCS. Furthermore, an asymmetric supercapacitor was assembled in combination with MXene (Ti_3_C_2_T_*x*_) as a negative electrode (denoted as TCX). As a result, the CF-NCS//TCX device exhibits a high areal capacitance of 48.6 mF cm^−2^ at 2 mA cm^−2^ of current density. We also report good specific capacitance of 54.57 F g^−1^ at 2 A g^−1^; in addition, the CF-NCS//TCX assembly delivers maximum areal and gravimetric energy density of 14.86 mWh cm^−2^ and 14.86 Wh kg^−1^ respectively. In contrast, the VG-NCS//TCX device showed improved cycling stability with 85% of capacitance retention over 5000 cycles owing to its highly porous structure and multiple conductive networks in the VG substrate and provides structural stability to NCS with fast ion diffusion. This experiment favors 2D MXene as a capacitive electrode that provides a replacement for carbon-based electrodes in asymmetric assembly with superior electrochemical performance. Hence, the hierarchical NCS structure grown on the various substrates in combination with MXene serve as a promising material for energy storage application.

## Abstract 2

Spinel metal sulfides have been investigated for a wide range of applications mostly in electrochemical energy storage owing to their better electronic conductivity and high reversible redox activity.

## Graphical abstract

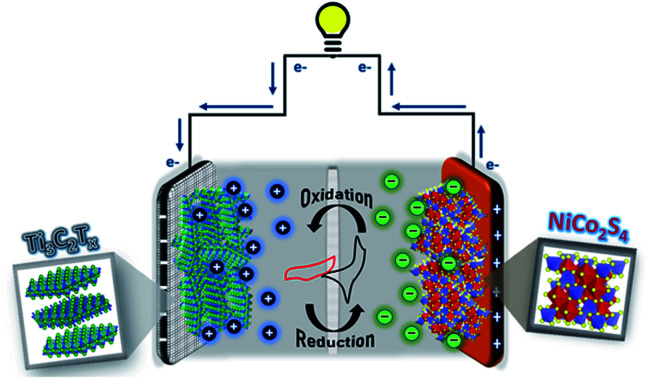

## Introduction

The growing demand for energy and perpetual extent of energy consumption have prompted a new challenge in the development of clean, green and highly efficient energy storage technologies. The electrochemical energy storage (EES) technology has grabbed massive attention due to the advancement in electrode fabrication, miniaturization and integrated designs. Supercapacitors (SCs) are considered as a potential candidate of EES owing to their high-power density, fast charge/discharge rate, long cycling life-time, low cost and most importantly environment-friendly features. Depending upon the underlying charge storage mechanism SCs are classified into two types: electric double layer capacitor (EDLC) in which the double layer arises due to accumulation of charges occurring at electrode/electrolyte interface electrostatically. Carbon-based materials with the high porous surface area are mainly used in commercial EDLCs. Whereas the fast faradaic (redox) process for the charge transfer is responsible for the pseudocapacitance. In general, SCs suffer low energy density attributed to the different charge storage mechanism that limits their practical applications. As a room for improvement, researchers worldwide are emphasizing more upon the advanced electrode materials along with designs and fabrication that can effectively enhance the overall electrochemical performance along with good cycling stability.^1–4^ Pseudocapacitors (PCs) can be considered as the potential system for EES as their performance is evaluated between Li-ion batteries and EDLCs which can be conveniently operated within the time duration of 10 s to 1 min.^5^ The critical factor in SCs is energy density and it is mainly ascribed by the charge storage mechanism with respect to corresponding kinetics at the electrode/electrolyte interface. The energy density can be improved by pushing up the adequate electron transfer and ease of access in the diffusion of electrolytic ions. Recently, transition metal sulfides (TMSs) have drawn impressive attention as PC material owing to its higher electronic conductivity, excellent theoretical capacitance, rich redox-active sites and less metal–anion bonding energy. Moreover, TMSs approximately exhibit capacitance more than two orders of magnitude higher than their analogous oxides and binary sulfides.^6,7^ Ternary nickel–cobalt sulfide (NiCo_2_S_4_) is known to be a dynamic PC electrode material due to high performance induced by rich redox-active sites. Spinel NiCo_2_S_4_ constitutes and initiates superior electrochemical response due to contribution from both cobalt and nickel ions which enhances the intrinsic electrical conductivity. NiCo_2_S_4_ exhibits high electronic conductivity with a value nearly ∼100 times more than its oxide counterpart *i.e.* NiCo_2_O_4_.^8,9^ However, TMSs deliver much less capacitance in actual application due to structural decomposition during long-range cycling performance.^10,11^ Also, the structural hierarchy and construction of electrodes impart more surface exposure, effective ionic conductivity, low contact resistance and shortened electron transfer path.^39^

To overcome the mentioned limitations, the direct construction of electrode material on a planar current collector can be beneficial in order to achieve advanced PC electrodes. Hierarchical architecture of the electrode material with superior electrochemical properties can be achieved by facile, easy and cost-effective synthesis approaches. Several current collectors such as metal foils/foams/mesh,^12,13^ carbon-based papers,^14,15^ textiles,^16^ cellulose,^17,18^ polymer-based^19^ and modified glass substrates^20^ have been explored so far as the electrode to grow the active materials. A 3D conducting Ni-foam is typically studied as a current collector for the growth/loading of electrode materials *via* facile synthesis routes owing to its desirable 3D network, high surface area, electronic conductivity and low cost. Its hydrophilic nature persuades better mass loading and opens up the channels for electrolytic penetration that smoothens the charge transport path.^21,22^ Cu-foil is widely considered as a skeleton that provides a smooth surface and large surface area for binder-free architectures. The surface oxidation of Cu-foil exists in two forms *i.e.*, Cu_2_O (Cu-i) and CuO (Cu-ii) respectively. Nanostructures are directly grown on Cu-foil substrate not only provides chemical stability to PC materials but also improves rapid ion transportation during the fast redox process.^23–25^ Several reports of decoration of PC materials on carbon-based materials have shown the improvement in the supercapacitive performance. Hierarchical 3D interconnected structure of vertical graphene nanosheets provides high surface area, remarkable thermal and electrical stability, non-agglomerated self-supporting structures and multiple conductive networks that stand as a backbone to PC materials with fast electron transport. Vertical graphene nanosheets support EDLC type behavior where structure remains intact even after undergoing very long-term charging/discharging cycles.^26,27^ Electrodes constructed using binder introduces “dead area” for energy storage due to aggregation of nanostructures and the electrolyte fails to penetrate below 20 nm drop of material. Therefore, effective binder-free routes of electrode fabrication are in great requirement.^28,42^

Recently discovered MXenes are considered as an emerging class of 2D materials for energy storage applications. MXenes include transition metal carbides, nitrides and carbonitrides with the chemical formula M_*n*+1_X_*n*_T_*x*_ (M-transition metals, X-carbon/nitrogen and T-surface terminal groups) that contributes to EDLC as well as pseudocapacitive nature.^29,30^ MXene reports exhibiting pseudocapacitance in acidic electrolytes. Meanwhile, they exhibit typical rectangular-shaped CV curves in aqueous salt and an ionic electrolyte which indicated EDLC capacitance arises due to cation intercalation inside MXene layers.^31,32^ The better electrochemical performance of next-generation SC is greatly dependent on achieving high energy density (*E*). The potential window of PC materials in the asymmetric assembly in an aqueous electrolyte can be widened by using MXene as a negative operating voltage window since carbon-based asymmetric devices are restricted to lower capacitance. In EDLC, low energy density is a critical affair. Energy density is mainly influenced by operating potential window (*V*) as it is given by the equation, *E* = 0.5*CV*^2^, that provides solution of tuning of “*V*” parameter to boost energy density in SC device in agreement with good power density, maximum capacitance and long cycle life.^33–35,41^

Here, we demonstrate a binder-free construction of NiCo_2_S_4_ (NCS) electrode structures *via* single-step electrodeposition technique on various substrates *viz.*, Cu-foil (CF), Ni-foam (NF) and vertical graphene nanosheets grown on carbon tape (VG) abbreviated as CF-NCS, NF-NCS and VG-NCS. The comparative investigation of electrochemical properties of as-prepared electrodes in two electrode symmetric and asymmetric assembly was studied using aqueous 0.5 M K_2_SO_4_ electrolyte by installing MXene as negative and NCS grown on CF, NF and VG substrate as a positive electrode. A high capacitance value of 167.28 F g^−1^ for CF-NCS was achieved in symmetric assembly. The CF-NCS//TCX asymmetric device exhibits maximum areal/gravimetric capacitance of 48.6 mF cm^−2^/54.57 F g^−1^ at 2 mA cm^−2^/2 A g^−1^ of current density within wider potential window of 1.4 V. It also delivers superior energy density of 13.22 mWh cm^−2^/14.86 Wh kg^−1^ at power density of 8291.29 mW cm^−2^/8197 W kg^−1^. In contrast, the VG-NCS//TCX device exhibit excellent cyclic stability with 85% of retention in capacitance over 5000 cycle due to interconnected network of VG substrate that provides structural stability. Thus, additive-free pseudocapacitor electrodes could bring up as a potential electrode material for electrochemical energy storage.

## Experimental

### Materials

A simple electrodeposition technique is involved in the synthesis of NiCo_2_S_4_ on various substrates. All the chemicals of analytical grade were used without further modification. The chemicals used were cobalt nitrate hexahydrate (Co(NO_3_)_2_·6H_2_O), nickel nitrate hexahydrate (Ni(NO_3_)_2_·6H_2_O), thioacetamide (C_2_H_5_NS), potassium chloride (KCl), potassium sulfate (K_2_SO_4_), ethanol LR, Nafion solution (5 wt% in mixture of lower aliphatic alcohols and 45% water), acetone LR, hydrochloric acid (HCl), Ni-foam (1.2 mm thickness, 1 cm diameter), Cu-foil (1 cm diameter), vertical graphene nanosheets on carbon tape (area 1 cm^2^), MAX-phase powder (Carbon, Ukraine Ltd. Particle size ≤ 200 micron), hydrofluoric acid (HF, 40%), distilled water.

### Preparation of MXene

MXene (Ti_3_C_2_T_*x*_) was successfully prepared *via* Al etching from Ti_3_AlC_2_ (MAX-phase powder) in HF at room temperature.^33,36^ Firstly, 25 ml of HF (40%) was transferred in a Teflon-lined container. A finely ground 500 mg of Ti_3_AlC_2_ powder was gradually added to the solution, carried under vigorous magnetic stirring for 24 hours at room temperature. The resulting suspension was then washed with distilled water several times till neutral pH was obtained. The precipitate was washed twice with ethanol and vacuum dried at 120 °C for 12 h the obtained powder was ground finely again and used for further characterization.

### Growth of VGN

The growth of hierarchical VG arrays on carbon tape was carried out by microwave plasma enhanced chemical vapor deposition method (MPECVD). The detailed procedure is reported by Ghosh Subrata *et al.* elsewhere.^26,37^ In a typical process, the growth of high-quality VG nanoarrays was carried out by placing the base in the reaction chamber in presence of plasma maintained at 300 W with operation pressure of 1.2 × 10^−3^ mbar at 800 °C in presence of pure CH_4_ and Ar as hydrocarbon source and carrier gas respectively. The grown VGN were further annealed for high quality and stable structure.

### Preparation of electrodes

A simple cost-effective method was employed for the fabrication of binder-free NCS nanostructures on three different substrates including Cu-foil (CF), Ni-foam (NF), VG Nanosheets on carbon tape (VG). VG substrate was used as received. NF and CF substrates were pretreated subsequently with acetone and distilled water followed by vacuum drying. The growth of NCS on various substrates was conducted *via* the electrodeposition technique. In a typical process, 0.16 M of cobalt nitrate hexahydrate (Co(NO_3_)_2_·6H_2_O), 0.08 M of nickel nitrate hexahydrate (Ni(NO_3_)_2_·6H_2_O), 0.48 M of thioacetamide (C_2_H_5_NS) and 0.08 M of potassium chloride was successively added in 80 ml distilled water. The mixture was sonicated for 10–15 minutes at room temperature. The chrono-amperometry technique was employed for the growth of NCS materials using a three-electrode cell set up where Pt wire and Ag/AgCl were used as counter and reference electrodes respectively.^38^ The pre-cleaned NF, CF and VG substrates were immersed in the solution that served as a working electrode. The solution temperature was maintained at 70 °C throughout the procedure. The deposition was achieved at −1.1 V potential for 180 seconds of coating duration. The electrodeposited NCS nanostructures were further vacuum dried at 60 °C for 12 h. The mass loading achieved was about 1.5 mg on CF and NF substrates and 1 mg on VG substrate respectively. The electrochemical applications of as-prepared electrodes were further analyzed.

For our negative electrode, (Ti_3_C_2_T_*x*_) MXene powder with 1 mg of active mass was dispersed in Nafion and ethanol solution (ratio 1 : 19) and sonicated for 15 min. Then the slurry was drop casted on cleaned Ni-foam and dried in a vacuum at 60 °C. The electrodes were pellet pressed with 5 tons pressure by a hydraulic pellet press.

### Materials characterization

The phase purity of as-prepared electrode materials was investigated by X-ray diffraction by X-ray diffraction (XRD, Rigaku Ultima IV having NI-filter for Cu-Kα radiation, *λ* = 0.1541 nm). The elemental and morphological analyses were performed by field emission scanning electron microscopy (FESEM, JEOL JSM-7100F, JEOL Ltd, Singapore). The electrochemical properties of prepared electrodes were investigated using an electrochemical workstation (CorrTest instrumental model-CS350, Wuhan, China).

## Results and discussion

### Electrode fabrication and characterization

**Fig. 1:**
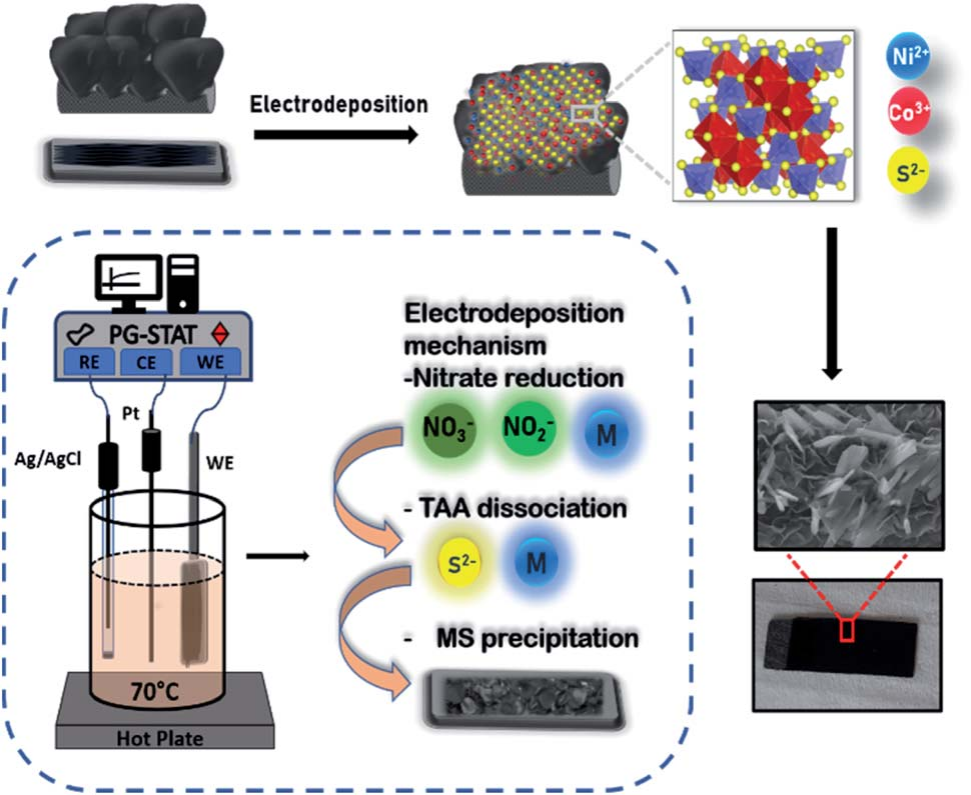
Schematic representation of electrodeposition process of NiCo_2_S_4_ electrode on vertical graphene substrate; inset: growth mechanism of NiCo_2_S_4_*via* electrodeposition method.

The electrochemical properties of symmetric and asymmetric devices in 0.5 M K_2_SO_4_ aqueous electrolyte using two-electrode Swagelok cell configurations were studied for both symmetric as well as asymmetric supercapacitor devices. To study redox mechanism and working potential range, three electrodes set up was employed where Pt wire and Ag/AgCl were used as counter and reference electrodes respectively. The stability test was estimated by GCD profiles from gravimetric capacitance value. Fig. 1 gives a schematic representation of the direct fabrication of NCS active materials on VG substrate. The formation of spinel structure due to metal ions from precursors after electrodeposition occurs on VG nanosheets. The irregular sheets of NCS grown of VG substrate are evident from the morphological characterization of the sample. Three steps are majorly involved for the formation of NCS structure on a substrate and the inset describes the steps involved. The fundamentals of the reactions are discussed as follows: (i) nitrate salt reduction-the polarized metal species from reduced nitrate ions (NO_3_^−^/NO_2_^−^) infuse to produce M(OH)_2_ species at cathode given by following reactions,^43,44^

1 NO_3_^−^ + H_2_O + 2e^−^ → NO_2_^−^ + 2OH^−^

2 NO_2_^−^ + 6H_2_O + 6e^−^ → NH_4_^+^ + 8OH^−^

The polarized metal species undergo electrolytic precipitation to hydroxide given by eqn (3) and (4) below,

3 M → M^2+^ + 2e^−^

4 M^2+^ + 2OH^−^ → M(OH)_2_

(ii) Simultaneously, the dissociation of thioacetamide produces H_2_S gas followed by a reversible reaction that rapidly accumulates the S^2−^ ions depending on the pH and temperature of the medium used for co-precipitation of metal sulfide species. The controlled mechanism of thioacetamide dissociation with thermal equilibrium is expressed by eqn (5) and (6),^45,46^

5 CH_3_CSNH_2_ + 2H_2_O → CH_3_COOH + NH_3_ + H_2_S

6 H_2_S ↔ HS^−^ + H^+^

7 HS^−^ ↔ S^−^ + H^+^

8 S^−^ + M^+^ → MS

(iii) Further, the produced M(OH)_2_ species magnetically react with S^2−^ generated by decomposition of thioacetamide to form MS. The product exhibit limited stability due to strong interparticle interaction than M(OH)_2_. The formation of MS could be proposed by following reaction,^47,48^

9 M(OH)_2_ + S^2−^ → MS(OH) + (OH)^−^

Moreover, the free M^+^ and OH^−^ react with S^2−^ for MS precipitation given by the following equation,

10 2S^2−^ + O_2_ + 2H_2_O + 2M → 2MS + 4OH^−^

**Fig. 2:**
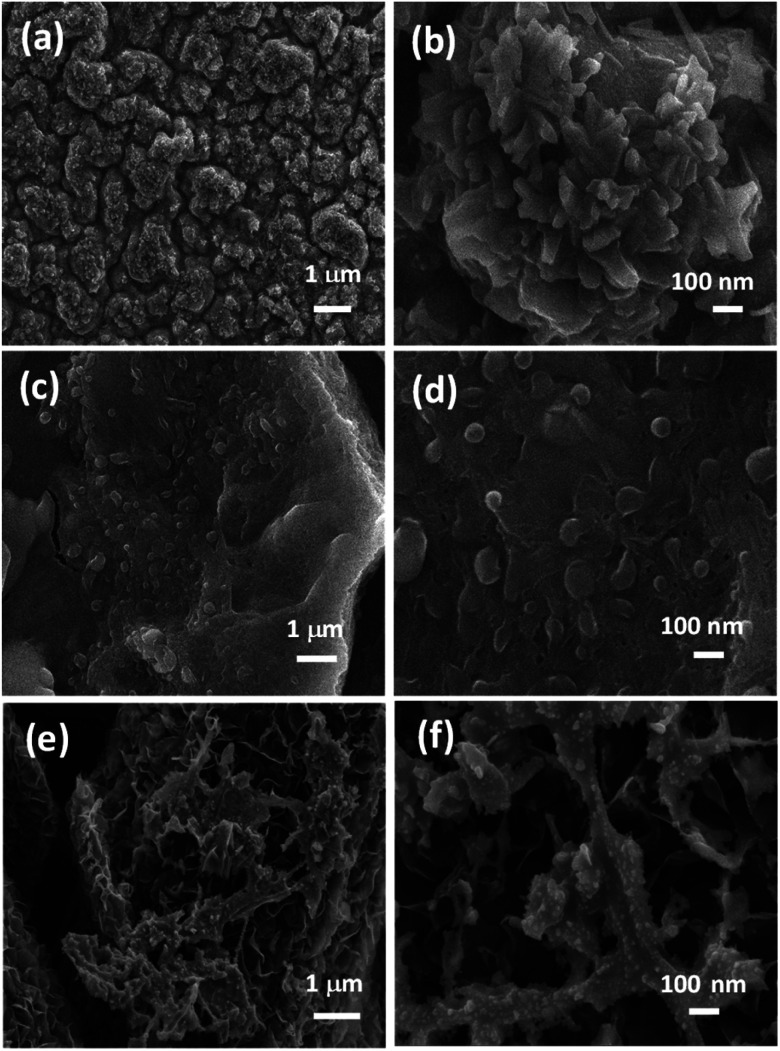
FESEM images of (a and b) growth of NCS on Cu-foil (CF); (c and d) growth of NCS on Ni-foam (NF); (e and f) growth of NCS on vertical graphene (VG) substrate.

Fig. 2 represents the morphological investigation of NCS nanostructures grown on various substrates obtained through field emission scanning electron microscopy technique. Fig. 2(a) displays a layered structure of CF-NCS. The homogeneously covered sheets appeared in a maze-type porous network assembled evenly in spherical symmetry resembling a flower-like appearance, shown by Fig. 2(b). As shown in Fig. 2(c and d) uniform distribution of closely packed Ni–Co–S spherically aligned structure formed due to uneven exchange of metallic S^2−^ atoms reacting slowly with Ni and Co ions to forms seed-like morphology.^40^Fig. 2(e and f) illustrates the cross-linked microarrays of NCS grown on VG substrate that enables adequate access to electrolytic ions due to interconnected network settled at the surface shaped by excessive deposition and mechanical expansion.^7^ The uniform deposition of porous NCS surface and cracks provides a channel to the flow of electrolytic ion diffusion. The homogeneously arranged VG interconnected porous nanosheets with abundant interspaces are resolved in Fig. S1(a).† Fig. S1(b)† gives a closer view of VG nanosheets. Fig. S1(c)† presents the perfectly MXene sheets derived after HF etching. The high-resolution micrograph shows prominent spacing between each layer that provides an enlarged inner surface which effectively imparts cation intercalation and diffusion paths for electrolytic ions, inset displays the crystal cells of MXene layers.^29^

**Fig. 3:**
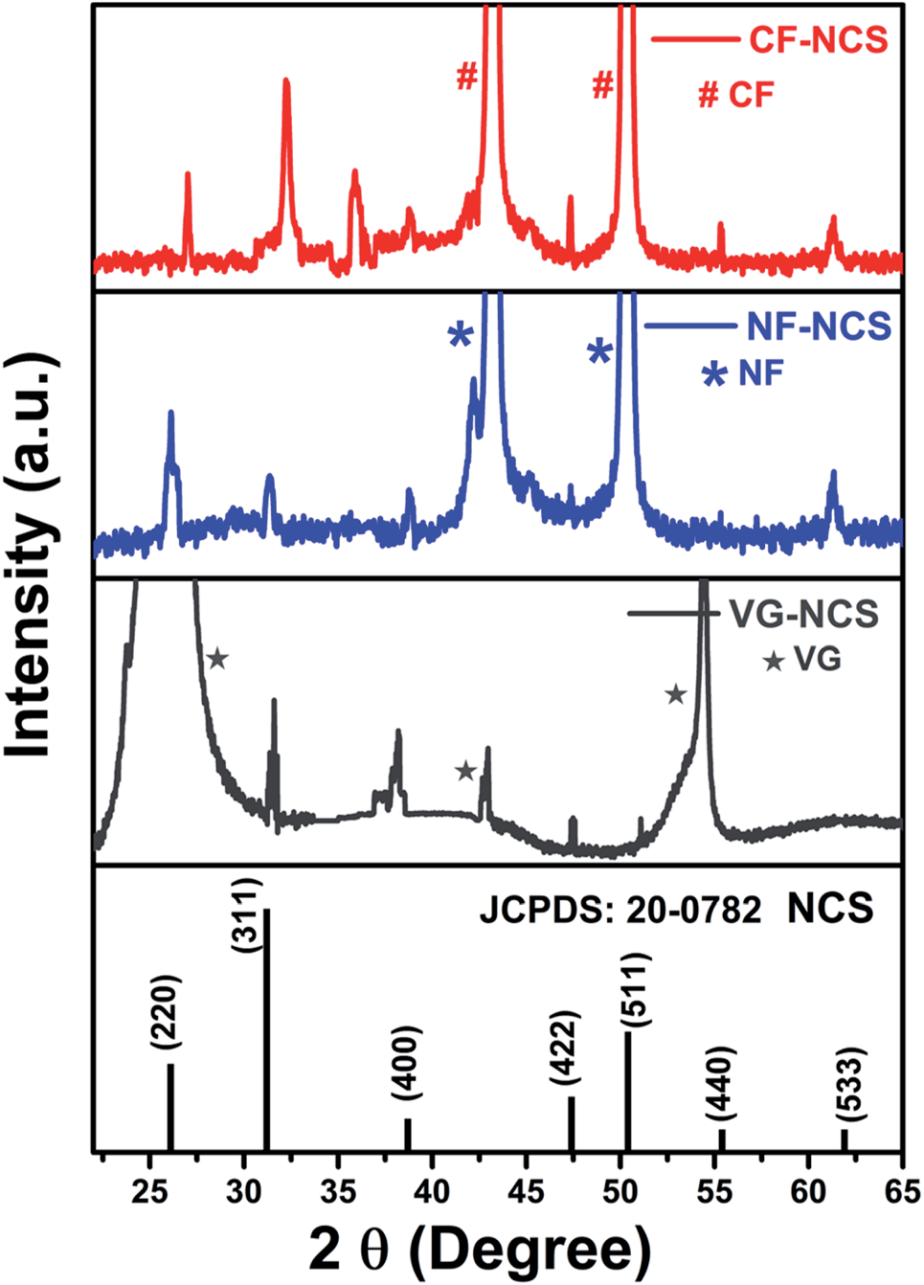
XRD analysis of (a) growth of NCS on Cu-foil (CF); (b) growth of NCS on Ni-foam (NF); (c) growth of NCS on vertical graphene (VG) substrate.

The phase purity of as-prepared electrodes was investigated by the X-ray diffraction technique illustrated in Fig. 3. Apart from strong peaks of Cu-foil, Ni-foam and VG substrate the representative peaks at low intensity of 2*θ* values 26.9°, 31.5°, 38.5°, 47.3°, 50.4°, 55.2°, 63.2° can be indexed to (220), (311), (400), (422), (511), (440) and (533) planes of the cubic phase of space group *Fd*3̄*m* (No. 227) of spinel NiCo_2_S_4_ [JCPDS card No. 20-0782]. Fig. S1(e)† shows the characteristic peaks of MXene after HF treatment. The shifting of 2*θ* values at 8.9° and 18.3° indicates the expansion in the MXene layer after Al etching. The hexagonal Ti_3_AlC_2_ belongs to *P*6_3_/*mmc* space group [JCPDS card No. 52-0875].^49^ In Fig. S2(f)† a sharp peak at 26.11° belongs to the (002) plane represents the high-quality VG nanosheets.^50^ The peak observed around 35° (2 theta) and other secondary peaks in CF-NCS could be attributed to α-phase of Ni and Co hydroxides (JCPDS-38-0.715), due to similar structural features differentiation between Ni and Co hydroxide is difficult. Ni_0.5_Co_0.5_(OH)_2_ phase, which demonstrates the phase transformation to NiCo_2_S_4_.^72^

### Electrochemical performance

**Fig. 4:**
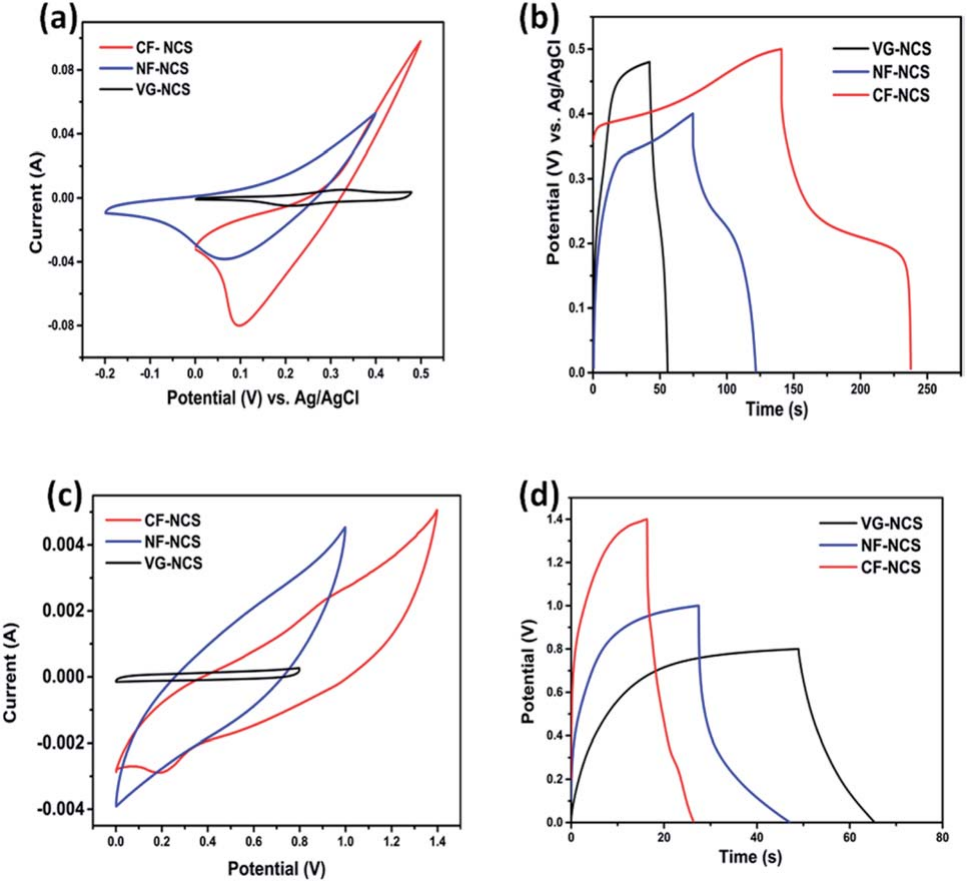
(a) the CV cures of NCS on CF, NF and VG substrates, (b) GCD curve of CF, NF and VG substrate in 3 electrode system (*vs.* AG/AgCl) in 0.5 M K_2_SO_4_ electrolyte, (c) the CV cures of NCS on CF, NF and VG substrates and (d) GCD curve of NCS on CF, NF and VG substrate.

The evaluation of electrochemical properties of as-prepared electrodes was investigated in symmetric as well as asymmetric assembly in 0.5 M K_2_SO_4_ aqueous electrolyte. Firstly, evaluation of redox activities and suitable voltage windows was carried out in a three-electrode configuration. Fig. S2† illustrates the CV and GCD profiles of all three binder-free CF-NCS, NF-NCS and VG-NCS electrodes that unveils the positive working window form −0.2 V to 0.5 V. The cyclic voltagramm shows the pair of distinct redox peaks particularly at lower scan rates clearly indicates high reversible redox reactions occurring at electrode/electrolyte interface. The ideal behavior of all the electrodes can be explained by well-maintained CV curves from lower to higher scan rates. The comparative characteristic faradaic property of all the electrodes recorded at 20 mV s^−1^ is displayed in Fig. S4(c).† For CF-NCS redox peaks appearing at 0.395 V and 0.22 V can be originated by Ni^2+^/Ni^3+^ and Co^2+^/Co^3+^/Co^4+^ species. Besides while electrodeposition Cu atoms in the Cu-foil produces highly conducting metallic arrays to build up the deepest contact with an electrode material that improves electronic conductivity which may contribute to the enhancement in electrochemical properties of CF-NCS.^25,51^ The redox peak around 0.39 V and 0.18 V was observed in NF-NCS are associated with the faradaic process of Ni^2+^/Ni^3+^ and Co^2+^/Co^3+^ transitions with electrolytic ions. The physical structure of Ni-foam enables more diffusion of electrolyte that leads to enhanced capacitance value.^52^ The reduced area under the CV curves in the case of VG-NCS at lower scan rates suggests the lesser contribution of the current collector while well-resolved redox peaks indicate the pseudocapacitive interactions from Ni and Co ions.^53^Fig. 4(a and b) gives the comparative analysis of all the electrodes. CF-NCS shows a remarkably larger integral surface area compared to other electrodes denotes the higher pseudocapacitance. In agreement with CV curves, GCD curves display a longer discharge time in CF-NCS than NF-NCS and VG-NCS. The notable enhancement of CF-NCS can be ascribed to the abundant porous channels, rich redox-active sites and contribution from both metal ions. During the faradaic process, the electrolytic ions interact only with the outer surface of the electrode whereas at lower scan rates diffusion and penetration through electrode material is more that pass more electrons through the current collector at the interface resulting into increased capacitance. The plausible redox reaction of NiCo_2_S_4_ in K_2_SO_4_ electrolyte is as follows,^9,54^

11 CoS_2*x*_ + *x*(SO_4_)^−^ ↔ (CoS_2*x*_–SO_4_)*x* + e^−^

12 NiS + *x*(SO_4_) ↔ (NiS–SO_4_)*x* + e^−^

Both metal ions participate in charge storage. The shifting of anodic and cathodic peaks towards higher and lower potentials could be ascribed to the polarization of the electrodes. The intact symmetry in the GCD plateau suggests the redox activity. The fast diffusion of SO_4_^2−^ ion on the active edges of NCS originates the excellent electrochemical properties.^55^

The electrochemical performance of the Ti_3_C_2_T_*x*_ electrode in a three-electrode configuration is displayed in Fig. S4(a and b).† Well-shaped quasi-rectangular CV curves indicate the foremost capacitive behavior recorded in negative potential within the range of 0 V to −0.8 V. A gradual drift in CV curves with the increasing scan rate due to less ion diffusion. Whereas rectangular shape at a lower scan rate implies higher ionic activity. The cation intercalation/deintercalation reaction arises due to the layered symmetry of the MXene sheets. The electrochemical kinetics at electrode/electrolyte interface can be expressed as,^10,34^

13 Ti_3_C_2_T_*x*_ + *x*K^+^ + *x*e^−^ ↔ K*x*Ti_3_C_2_T_*x*_

14 Ti_3_C_2_ + *x*K^+^ + *x*e^−^ ↔ K*x*Ti_3_C_2_

Here, *x* represents the number of electrons involved. The spontaneous intercalation of K^+^ ion between 2D MXene layers is implied by the peak broadening which dominated EDL capacitance due to the presence of functional groups (–O, –OH and –F). The linear charge/discharge response of GCD cycles from 0 V to −0.4 V arises due to EDL capacitance whereas the non-linearity appearing at −0.4 V to −0.8 V specifies pseudocapacitance. The combined characteristics of the capacitive and faradaic process arise due to a change in the oxidation state of the outer Ti layer of MXene.^56–58^

### Electrochemical performance of symmetric and asymmetric devices

**Fig. 5:**
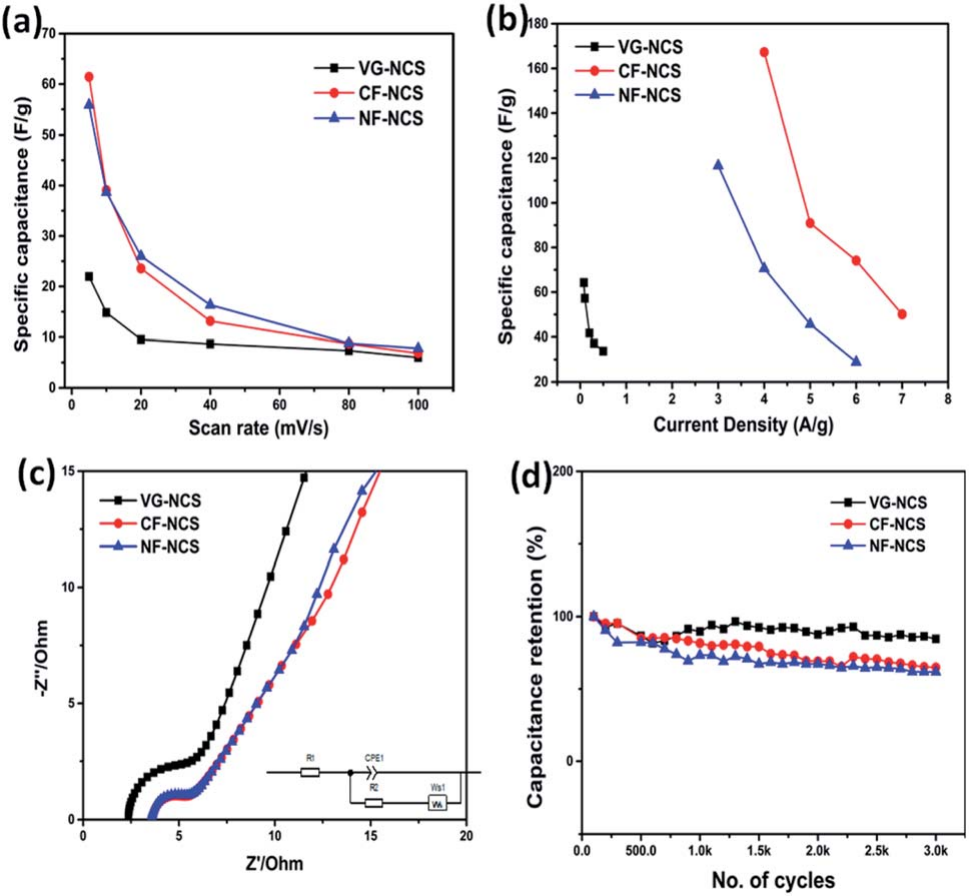
(a) graph of specific capacitance (F g^−1^) *vs.* scan rate (mV s^−1^), (b) graph of specific capacitance (F g^−1^) *vs.* current density (A g^−1^), (c) Nyquist plot of NCS on CF, NF and VG substrate (inset: simulated circuit) and (d) graph of specific cycling stability of all electrodes tested in symmetric 2 electrode cell assembly using Swagelok cell.

The practical applications of our as-prepared electrodes were further evaluated in the symmetric and asymmetric configuration using the two-electrode Swagelok cell (Whatman filter paper as separator). First, the NCS electrodes decorated on CF, NF and VG substrates were examined in symmetrical assembly. Fig. 4(c and d) compares the electrochemical performance of symmetric devices. The typical CV curves recorded at 100 mV s^−1^ are displayed in Fig. 4(c). CF-NCS shows excellent capacitive behavior compared to NF-NCS and VG-NCS devices. The enlarged voltage window and distinctly larger area under the curve imply the high performance of CF-NCS, which could be attributed to purely metallic Cu interstitial species scraped on Cu-foil during the electrodeposition process of NCS growth which provides a rapid response to reversible redox reactions. The typical CV curves of all the symmetric electrodes obtained at various scan rates are shown in Fig. S3.† The variation in specific capacitance with respect to different scan rates of all the electrodes is displayed in Fig. 5(a). Further GCD analyses were carried out in the same potentials as CV profiles. The competitive GCD response is shown in Fig. 4(d). Fig. 5(b) shows the specific capacitance calculated from GCD data. Here also CF-NCS shows maximum specific capacitance of 167.28 F g^−1^ at 4 A g^−1^ of current density. NF-NCS exhibit 116.62 F g^−1^ at 3 A g^−1^ and VG-NCS gives 64.26 F g^−1^ at 0.08 A g^−1^ respectively. Fig. 5(b) plots the relationship between specific capacitance and current density. The specific capacitance decreases with increasing current density values. Furthermore, stability of symmetric devices was tested values, VG-NCS displays superior stability with capacitance retention of 81% over 3000 cycles at 20 A g^−1^. CF-NCS and NF-NCS exhibit 66% and 61% of capacitance retention over 3000 cycles (Fig. 5(c)). The order of specific capacitance calculated from CV and GCD cycles shows a similar trend to that of capacitance values obtained by three-electrode data. Fig. S3(g)† provides the Ragone plot of all symmetric devices. In comparison, CF-NCS delivers a high energy density of 11.38 Wh kg^−1^ at 8550 W kg^−1^ of power density. Comparative performances of symmetric as well as asymmetric devices based on NiCo_2_S_4_ material are displayed in Table 1. In order to obtain further insights, electrochemical impedance spectroscopy (EIS) was employed. The charge transfer kinetic of all the electrodes was performed at open circuit potential at a frequency range between 0.01 Hz to 100 kHz with 5 mV of potential. Fig. 5(e) expresses the Nyquist plots. Inset shows an equivalent circuit used to simulate experimental results. The component *R*_s_ is the resistance given by the real axis corresponds to electrolytic resistance and intrinsic resistance of electrode material. The diameter of the semicircle at a higher frequency region indicates the charge transfer resistance denoted by *R*_ct_. The linear part corresponds to Warburg impedance (*Z*_w_) represents the diffusion of electrolyte ions at the high-frequency region resulting from ion diffusion and transport in electrolyte. *C*_dl_ represents double layer capacitive element. Interestingly, not much difference between *R*_ct_ values of CF-NCS and NF-NCS was observed around ∼1.9 Ω and 2.2 Ω respectively. *R*_ct_ value of VG-NCS was calculated around ∼3.5 Ω yet shows low solution resistance (∼2.4 Ω). The low *R*_s_ value in VG-NCS ensures the effective ion diffusion at the electrode/electrolyte interface. Low ohmic resistance in CF-NCS implies better reaction kinetics.^59,60^ The *R*_s_ value is combined resistance at the interface between the current collector and electrolyte. The bulk resistance of foil and foam substrates is reported to be almost the same. The higher *R*_s_ value arises due to higher surface area and thinner sides in both Cu-foil and Ni-foam substrates.^61,62^

**Table 1:** Comparison of the energy storage performance of the supercapacitors based on nickel cobalt sulfides in symmetric and asymmetric assembly

Material	Specific capacitance (F g^−1^)	Areal capacitance (mF cm^−2^)	Energy density (Wh kg^−1^)	Power density (W kg^−1^)	Areal energy density (mWh cm^−2^)	Areal power density (mW cm^−2^)	Cycling stability (%/cycles)
CF-NCS//CF-NCS	167.28	—	11.38	8550	—	—	66/3000
NF-NCS//NF-NCS	116.62	—	4.5	5170	—	—	61/3000
VG-NCS//VG-NCS	64.26	—	1.5	2177	—	—	81/3000
CF-NCS//TCX	54.57	48.6	14.86	28870	13.22	25412	79/5000
NF-NCS//TCX	49	45	8.76	9051	8.74	8050	77/5000
VG-NCS//TCX	47	42.83	8.61	2555	8.57	2290	85/5000

**Fig. 6:**
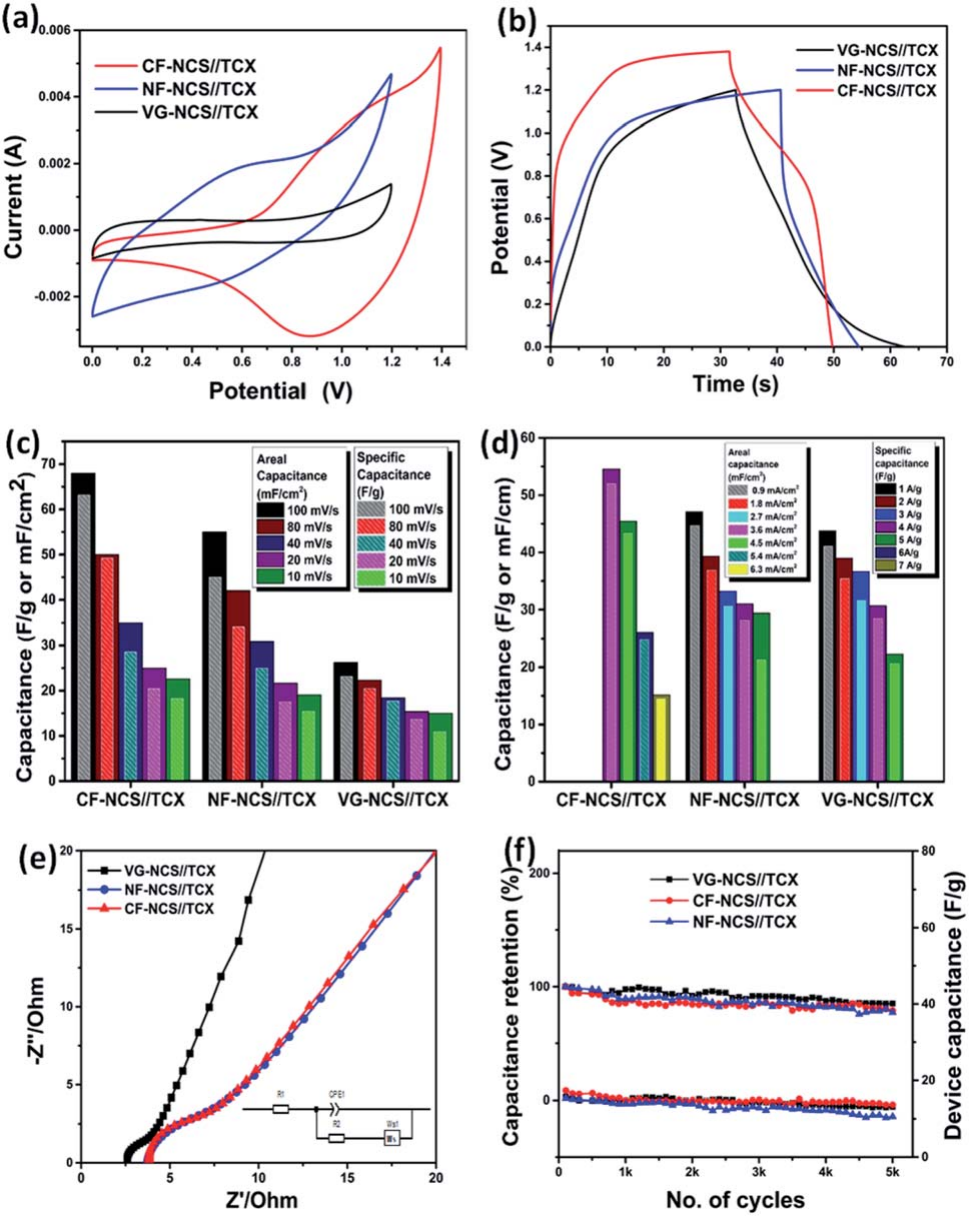
(a) the CV cures of NCS on CF, NF and VG substrates, (b) GCD curve of CF, NF and VG substrate in asymmetric assembly, electrochemical characterization (c) graph of areal capacitance (mF cm^−2^) and specific capacitance (F g^−1^) *vs.* scan rate (mV s^−1^), (d) graph of areal capacitance (mF cm^−2^) *vs.* current density (mA cm^−2^) and specific capacitance (F g^−1^) *vs.* current density (A g^−1^), (e) Nyquist plots of all electrodes; inset-equivalent circuit for Nyquist plot fitting, (f) graph of specific cycling stability of all the electrodes over 5000 cycles.

Along with superior performance, asymmetric devices exhibit excellent cyclic stability. All-pseudocapacitive-based asymmetric devices hold excellent electrochemical properties but fail to provide high-rate cycling performance in aqueous electrolytes. Hence asymmetric devices based on the combination of EDLC and pseudocapacitor are mostly preferred. In aqueous electrolytes, redox-rich kinetics appear at positive potentials weakens the structural stability. The physical and chemical stability of EDLC materials can have a dramatic impact on asymmetric devices. Here two different mechanisms of electrode materials participate in maximizing the operational voltage window of full asymmetric devices. With energy density prospective asymmetric devices offer comprehensive applications for EES with advanced overall performance.^63^ In K_2_SO_4_, the overall performance improves due fastest migration speed of K^+^ hydrated ions owing to its smaller hydrated ionic radius (3.31 Å) that enables access to inner pores of electrodes with the shortest relaxation and high ionic conductivity which overall progresses the electrochemical properties. The sulfate anions (SO_4_^2−^ with hydrated radius 7.3 Å) enables to set stable voltage window at higher potential as a lower concentration of OH^−^ ions shifts electrolysis process to higher potential in neutral electrolyte.^64,65^ The asymmetric device was assembled using diverse substrate-based NCS as a positive electrode and MXene as a negative electrode respectively. The CV test was conducted in order to adjust the operating voltage window for a single device with respect to the different charge storage mechanisms. Fig. S4(c)† displays the optimized positive and negative electrodes in 3 electrode configurations. The charge balances *Q*_+_ = *Q*_−_ were obtained for the appropriate mass balance of electrodes. The potential window was estimated from the CV that varies from 1.2 V to 1.4 V. The characteristic CV curves of the combined capacitive and redox process are displayed in Fig. 6(a).

The dominant faradaic charge storage nature was observed from the CV profile of the CF-NCS//TCX device at 100 mV s^−1^. The alteration in the peaks may rise due to the outer Ti layer of MXene and the transition of Ni/Co ions. CF-NCS//TCX device shows a maximum potential window of 1.4 V whereas NF-NCS//TCX and VG-NCS//TCX adjust to 1.2 V. The non-ideal behavior of CF-NCS//TCX is displayed in Fig. S5(c)† where well-maintained symmetry at different scan rates suggests the excellent reversibility. The dominating capacitive nature of the NF-NCD//TCX device depicts the ideal nature maintained by CV recorded at all scan rates, displayed in Fig. S5(a).† The CV analysis at various scan rates of VG-NCS//TCX indicates the ideal capacitive nature shown in Fig. S5(b).† The symmetric outline of charge/discharge curves with distorted rectangular shapes demonstrates the dominating faradaic nature of CF-NCS//TCX which is in better agreement with recorded CV curves. The GCD profiles of NF-NCS//TCX and VG-NCS//TCX at various current densities with longer discharge time shows linear behavior indicating capacitive response similar to the CV analysis. The negligible IR drop suggests unusual electronic conductivity from both electrodes. As expected, our CF-NCS//TCX device exhibits high specific and areal capacitance of 54.57 F g^−1^ and 48.6 mF cm^−2^ at a current density of 2 A g^−1^ and 2 mA cm^−2^ respectively (Fig. 6(a)). The specific and areal obtained from CV cycles at different scan rates is displayed in Fig. 6(c) and the specific/areal capacitance is calculated from GCD profiles at various current densities based on total mass and reduced area on both the electrodes is displayed in Fig. 6(d). The comparison of electrochemical performances of NCS on various substrates in symmetric as well as the asymmetric assembly is given in Table 1. For further clarification, EIS measurements were performed. The Nyquist plot is shown in Fig. 6(e). The low charge transfer resistance (*R*_ct_) and solution resistance (*R*_s_) were observed in the VG-NCC//TCX device (*R*_s_ ∼ 2.57 Ω and *R*_ct_ ∼ 0.99 Ω). The reduction in *R*_ct_ value of VG-NCS//TCX computes nearly capacitive performance. CF-NCS//TCX and NF-NCS//TCX device exhibit nearly equal charge-transfer and solution resistance (for CF-NCS//TCX-*R*_ct_ ∼ 4.57 Ω, *R*_s_ ∼ 3.85 Ω and for NF-NCS//TCX-*R*_ct_ ∼ 4.52 Ω, *R*_s_ ∼ 3.75 Ω) respectively. Warburg impedance at lower frequency region indicates faster ion diffusion than symmetrical devices initiated due to joint characteristics of both the electrodes.

**Fig. 7:**
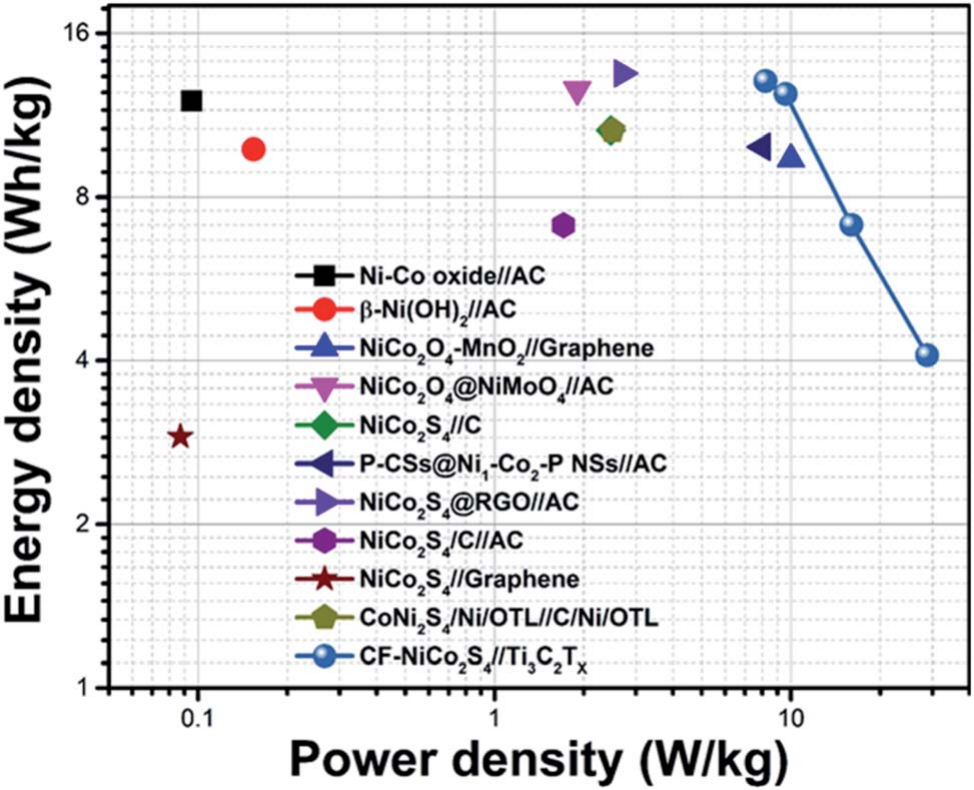
Comparative Ragone plot of specific energy density (Wh kg^−1^) *vs.* power density (W kg^−1^) of all the supercapacitor electrodes of Ni–Co based asymmetric devices.

Cycling stability is another crucial factor that reflects the practical significance of electrodes. The cyclic performance of CF-NCS//TCX and NF-NCS//TCX was obtained by GCD profiles over 5000 cycles at 30 A g^−1^ and VG-NCS//TCX at 20 A g^−1^ respectively. The capacitance retention of 79% and 77% was observed in CF-NCS//TCX and NF-NCS//TCX. Apparently, VG-NCS//TCX favors 85% of retention in capacitance about 5000 cycles. Aforementioned, the improved stability can be ascribed to the porous network of VG nanosheets that effectively gives structural support. The rate performance of asymmetric devices with MXene can be impressively enhanced due to the rapid diffusion of electrolyte in between the channels of layered MXene sheets.^65,66^ No significant degradation in capacitance reveals negligible changes in the structure of electrode material. Fig. S6† depicts the Ragone plot of all asymmetric devices that serves another practical purpose. The related specific and areal energy density and power density with respect to active electrode mass and area were achieved through GCD measurements. CF-NCS//TCX device delivers superior energy density of 14.86 Wh kg^−1^ and 13.22 mWh cm^−2^ at power density of 8197 W kg^−1^ and 8291.29 mW cm^−2^. NF-NCS//TCX device exhibits a maximum energy density of 8.76 Wh kg^−1^ and 8.74 mWh cm^−2^ at a power density of 2298 W kg^−1^ and 2295 mW cm^−2^. The respective energy density of VG-NCS//TCX was calculated to be 8.61 Wh kg^−1^ and 8.57 mWh cm^−2^ at a power density of 306 W kg^−1^ and 273 mW cm^−2^ respectively. Fig. 7 represents comparative Ragone plot of asymmetric devices based on Ni–Co based asymmetric supercapacitors with existing literature *e.g.* Ni–Co oxide//AC,^67^ β-Ni(OH)_2_//AC,^68^ NiCo_2_O_4_–MnO_2_//graphene,^69^ NiCo_2_O_4_@NiMoO_4_//AC,^70^ NiCo_2_S_4_//C (porous carbon),^71^ P-CSs@Ni_1_–Co_2_–P-NSs//AC,^72^ NiCo_2_S_4_@RGO//AC,^73^ NiCo_2_S_4_/C//AC,^74^ NiCo_2_S_4_//graphene,^75^ CoNi_2_S_4_/Ni/OTL//C/Ni/OTL^76^ and with our CF-NiCo_2_S_4_//Ti_3_C_2_T_*x*_. The overall performance of all asymmetric devices indicates good reversibility and high electronic conductivity including practical applications. The self-restacking feature of 2D MXene ensures the high-rate capability. The extreme redox-active sites in NCS offer the diffusion-controlled process and MXene represents capacitive controlled nature along with structural stability.

The unique morphology, extraordinary surface area and interconnected channels in CF-NCS and 2D MXene allow abundant access to electrolytic ions. The additive-free architecture provides a more effective and smoother electron transfer to the current collector. In addition, Cu species can intensively enhance the electrochemical activity as Cu nanostructures are reported to provide outstanding conductivity to the corresponding structures. The flexible Cu-foils provide a structural backbone for NCS structure. Also, the oxidation of Cu-foil may form CuO arrays on the surface during the electrodeposition process that provides effective exposure of faradaic active sites of NCS species with a synergistic effect that boosts the electrochemical activity.^77^ Also, mass loading plays an important role in supercapacitor performance, optimized mass loading significantly influences the energy and power density whereas, the thinner mass loading shows insufficient energy density.^78–80^ Optimum mass loading in CF-NCS and NF-NCS shows better electrochemical performance but lower mass-loading in VG-NCS exhibits lower energy density. The Ni-based substrates may deprive the electrochemical properties due to the formation of hydroxide species that leads to depleting the conductivity.^40^ The VG-based arrays extend chemical wettability and prevent aggregation of active electrode materials resulting in improved rate capability. The porosity and binder-free architecture report fast ion/electron transport and adequate surface to electrode/electrolyte interface. Thus, our work may provide an alternative for capacitive electrode material (MXene). The new approach to construct binder-free Ni–Co–S-based nanostructures with superior electrochemical properties could be served as a promising electrode material for advanced energy storage applications.

## Conclusions

In conclusion, we have demonstrated a simple strategy to fabricate binder-free active NiCo_2_S_4_ nanostructures directly on suitable substrates *viz.*, Cu-foil, Ni-foam and VG nanosheets. The successive construction of NCS offers extensive conductive networks ensuring high electronic conductivity. As a result, symmetric CF-NCS devices exhibit a high specific capacitance of 167.28 F g^−1^ at 4 A g^−1^ with maximum energy and power density of 11.38 Wh kg^−1^ and 8550 W kg^−1^ respectively. VG-NCS shows improved cycling stability owing to its open porous network (81%/3000 cycles) compared to NF-NCS and CF-NCS which follows CF-NCS > NF-NCS > VG-NCS trend. The asymmetric device was assembled by combining MXene and our NCS electrodes as negative and positive electrodes. In the case of the asymmetric supercapacitors, superior performance was achieved for CF-NCS//TCX device. The device exhibits excellent specific capacitance of 54.57 F g^−1^ at 2 A g^−1^ and areal capacitance of 48.6 mF cm^−2^ at 2 mA cm^−2^ respectively. The device also delivers notable energy density of 14.86 Wh kg^−1^ and 13.22 mWh cm^−2^ at power density of 8197 W kg^−1^ and 8291.29 mW cm^−2^ with better cycling stability (79%/5000 cycles). In contrast, the VG-NCS//TCX device exhibits an impressive cyclic performance of 85% up to 5000 cycles. The asymmetric device also follows a similar trend of CF-NCS//TCX > NF-NCS//TCX > VG-NCS//TCX. The mentioned properties of our as-prepared electrodes demonstrate that it can be employed for the fabrication of miniaturized device for future electronics.

## Conflicts of interest

There are no conflicts to declare.

## Supplementary Material

RA-012-D2RA00991A-s001
