# Supplementary material for: High performance asymmetric supercapacitors based on Ti3C2Tx MXene and electrodeposited spinel NiCo2S4 nanostructures
Source: RSC Adv. 2022 Apr 7;12(17):10788–99. doi: 10.1039/d2ra00991a (PMC8988171; doi:10.1039/d2ra00991a)
Supplement: RA-012-D2RA00991A-s001 [file RA-012-D2RA00991A-s001.pdf]

Electronic Supplementary Material (ESI) for RSC Advances.
This journal is © The Royal Society of Chemistry 2022

Electronic Supplementary Information

High performance asymmetric supercapacitors based on $Ti_3C_2T_x$ MXene and electrodeposited spinel $NiCo_2S_4$ nanostructures

Mansi Pathak[1], S.R. Polaki[2] and Chandra Sekhar Rout[1*]

[1]Centre for Nano and Material Science, Jain University, Jain global campus, Jakkasandra, Ramanagaram, Bangalore - 562112, India.

[2]Surface and Nanoscience Division, Materials Science Group, Indira Gandhi Centre for Atomic Research-Homi Bhabha National Institute, Kalpakkam, Tamil Nadu 603102, India

Email: csrout@gmail.com, r.chandrasekhar@jainuniversity.ac.in (CSR)

Electrochemical measurements:

The specific capacitance calculated using cyclic voltammetry,

$$Csp = \frac{\int I(V)dv}{[2m\ s\ \Delta V]}$$

Where, m is the mass of electrode deposited on substrate, s is the scan rate, ΔV is the potential window and ∫I(V) dV represents area under the curve.

Specific capacitance calculated using charge-discharge cycle,

$$Csp = \frac{I\Delta t}{m\Delta V}$$

Where, I is the current, m is the mass of electrode, Δt is discharge time and ΔV is the potential window. Current/mass is given by current density i.e. A/g and discharge time/ potential window can be obtained by the slope of the discharging curve from galvanostatic charge-discharge plot (Δt/ΔV).

Energy density of symmetric cell is calculated using following formula,

$$Ed = \frac{1}{2}Csp\ x(\Delta V)^2$$

Where, Csp is a specific capacitance calculated from GCD and ΔV is the potential window. (Wh/kg)

Power density of symmetric cell is calculated by using following formula,

$$Pd = \frac{Ed\ x\ 3600}{\Delta t}$$

Where, Ed is the energy density and Δt is the discharging time. (W/kg) [13,39].

To achieve the charge balance Q+ = Q−, a mass balance is required and given by following eq,

$$\frac{m_+}{m_-} = \frac{C_- * \Delta V_-}{C_+ * \Delta V_+}$$

The average mass ratio between the two electrodes m+/m− can be adjusted to get the optimal performance of an asymmetric supercapacitor.[40].

Areal capacitance calculated using cyclic voltammetry,

$$Ca = \frac{\int I(V)dv}{[A\ s\ \Delta V]}$$

Where, A is the active area of the electrode, s is the scan rate, ΔV is the potential window and ∫I(V) dV represents area under the curve. Unit is given by (mF/cm^2).

Areal capacitance calculated using charge-discharge cycle,

$$Ca = \frac{I\Delta t}{A\Delta V}$$

Where, I is the current, A is the geometrical area of electrodes, Δt is discharge time and ΔV is the potential window. Current/geometrical area of the electrode is given by current density i.e. mA/cm^2 and discharge time/ potential window can be obtained by the slope of the discharging curve from galvanostatic charge-discharge plot (Δt/ΔV), unit is given by (mF/cm^2).

Energy density of asymmetric cell is calculated using following formula,

$$Ed = \frac{1}{2} Ca\ x (\Delta V)^2$$

Where, C_a is a specific capacitance calculated from GCD and ΔV is the potential window. ($mWh/\ cm^2$)

Power density of asymmetric cell is calculated by using following formula,

$$Pd = \frac{Ed\ x\ 3600}{\Delta t}$$

Where, Ed is the energy density and Δt is the discharging time. (mW/ cm^2). [41,42].

Figure S1

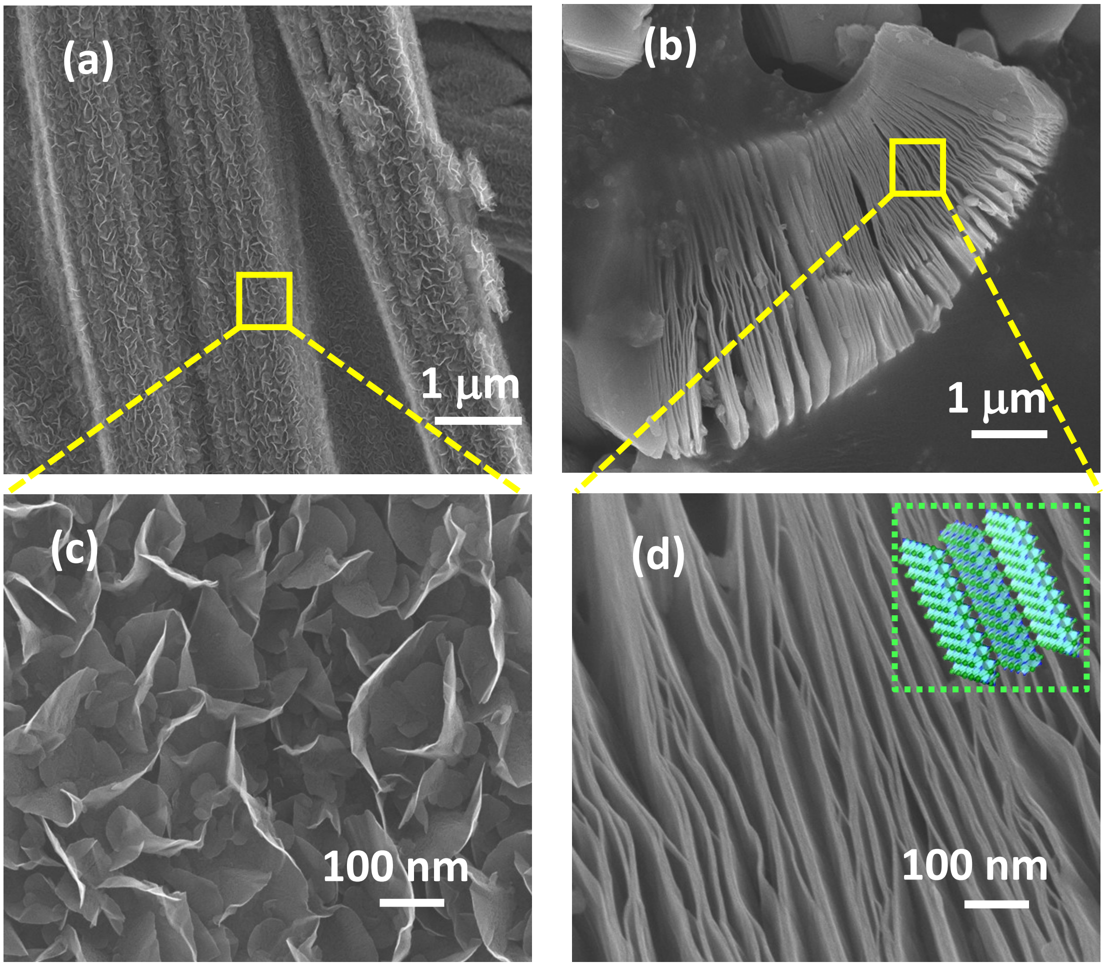

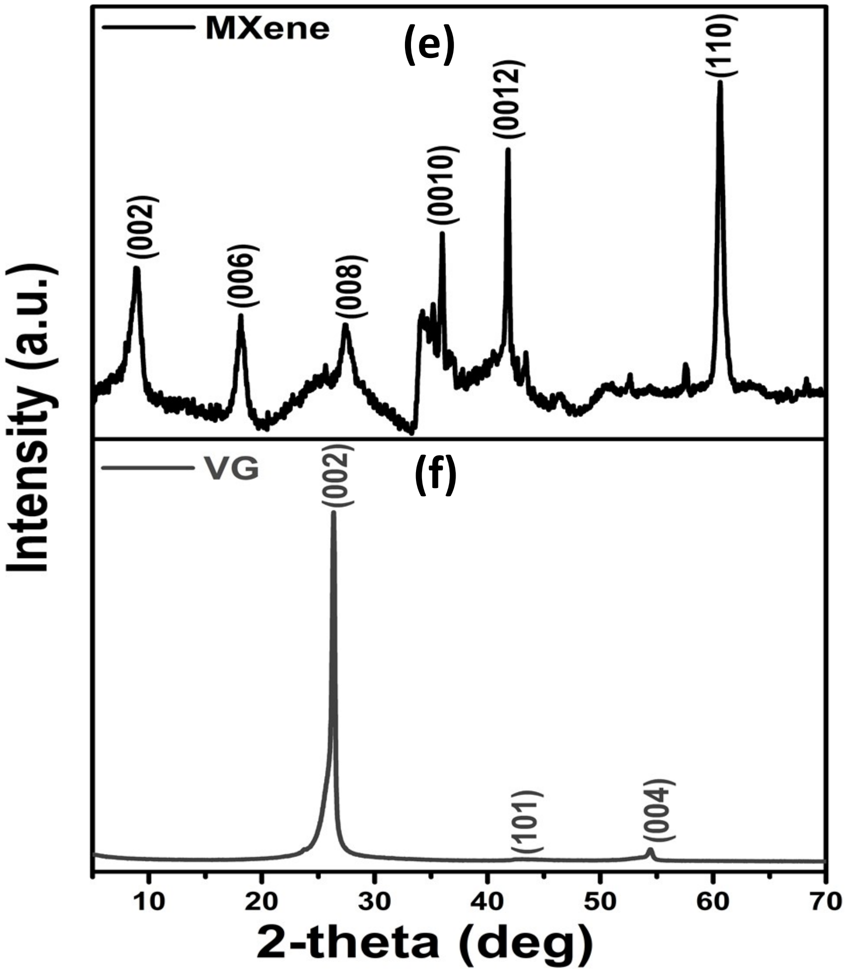

Fig-S1: FESEM image of (a) vertical graphene (VG) (b) magnified image VG, (e) MXene Sheets after HF etching and (d) image of HF etched MXene sheets at high magnification. Inset shows 2D sheets like structure after HF etching; XRD pattern of (e) MXene Sheets after HF etching, (f) vertical graphene (VG).

Figure S2

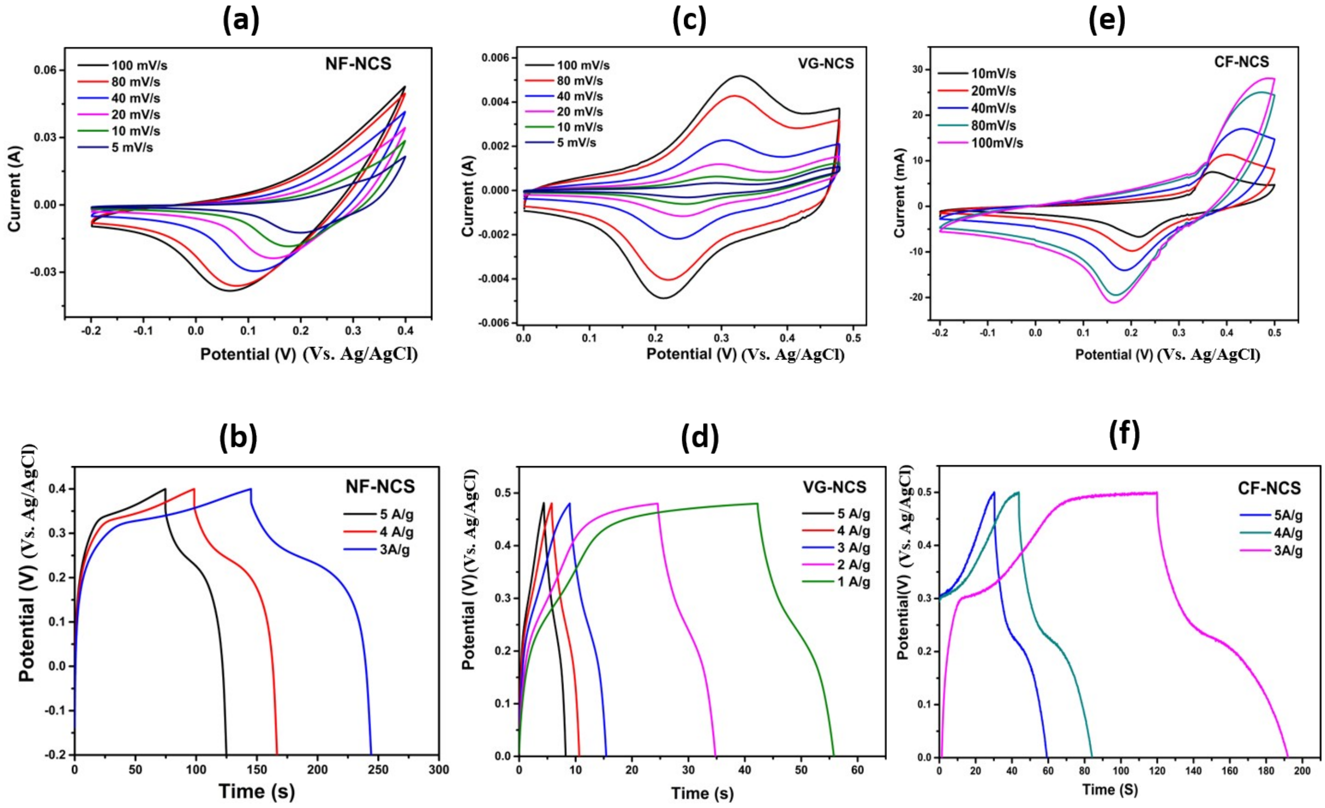

Fig-S2: (a-b) the CV and GCD curves of NCS on substrate, (c-d) CV and GCD curve of NCS on VG substrate; (e-f) CV and GCD curve of NCS on CF substrate- in 3 electrode cells set up (Vs. Ag/AgCl) 0.5M K_2SO_4 electrolyte

Figure S3

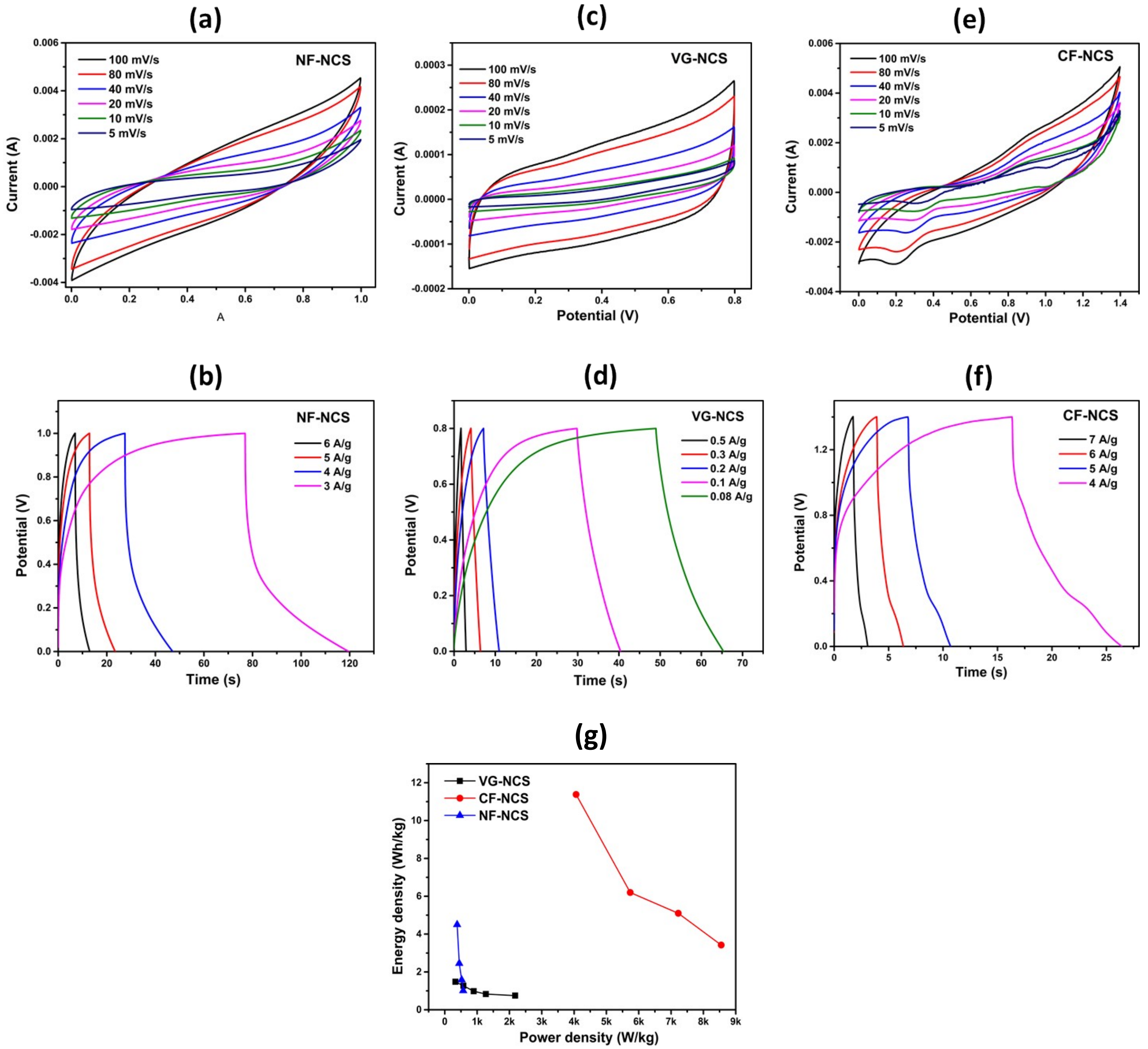

Fig-S3: (a-b) the CV and GCD curves of NCS on substrate, (c-d) CV and GCD curve of NCS on VG substrate; (e-f) CV and GCD curve of NCS on CF substrate and (g) Ragone plots of all the supercapacitor electrodes of NCS grown on VG, CF and NF substrate.

Figure S4

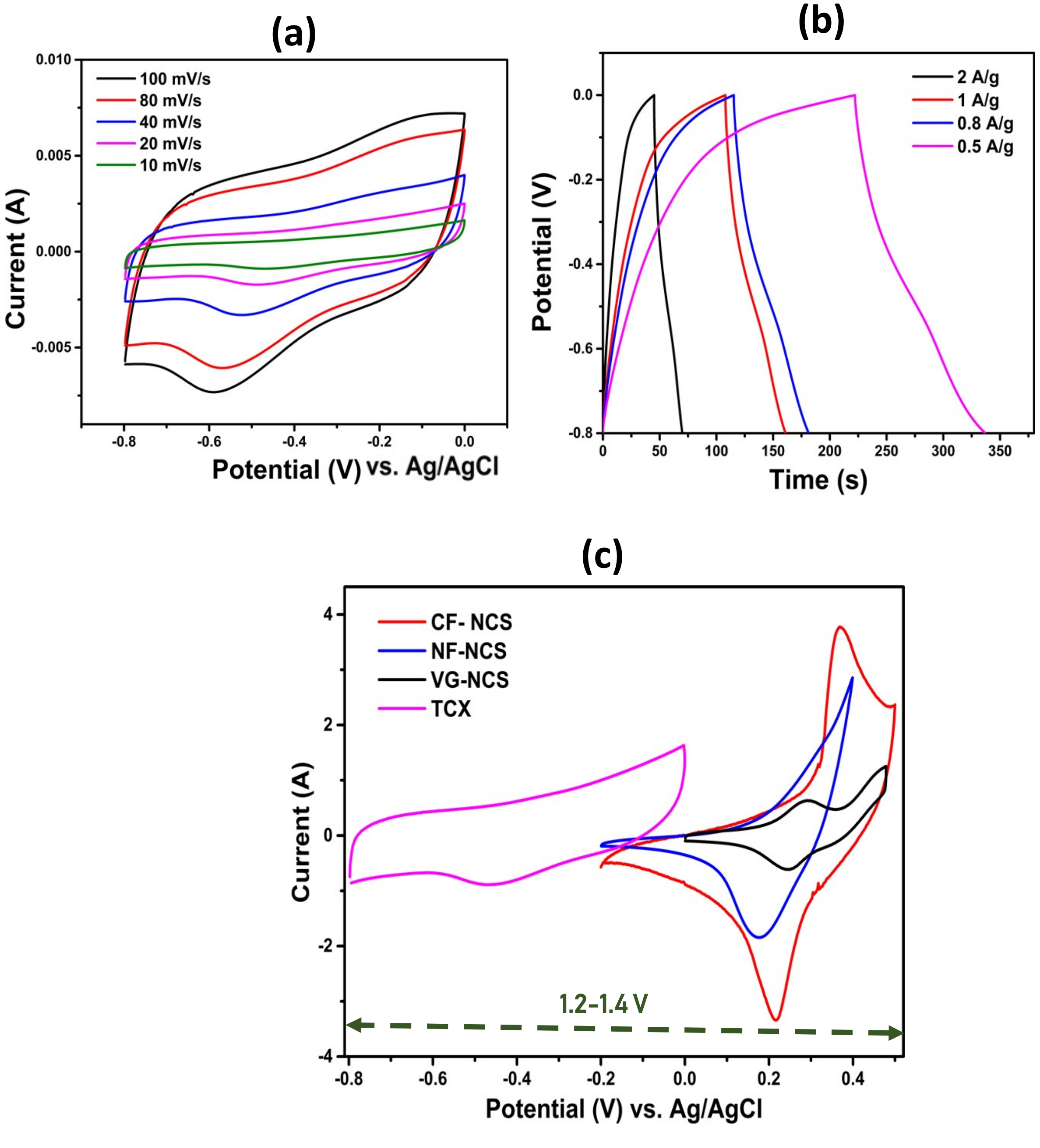

Fig-S4: (a) the CV cures of $Ti_3C_2T_x$, (b) GCD curve of $Ti_3C_2T_x$ in 3 electrode system (Vs. AG/AgCl) and (c) comparative CV cycles of NCS grown on CF/NF/VG substrates and $Ti_3C_2T_x$ in 0.5M K_2SO_4 electrolyte

Figure S5

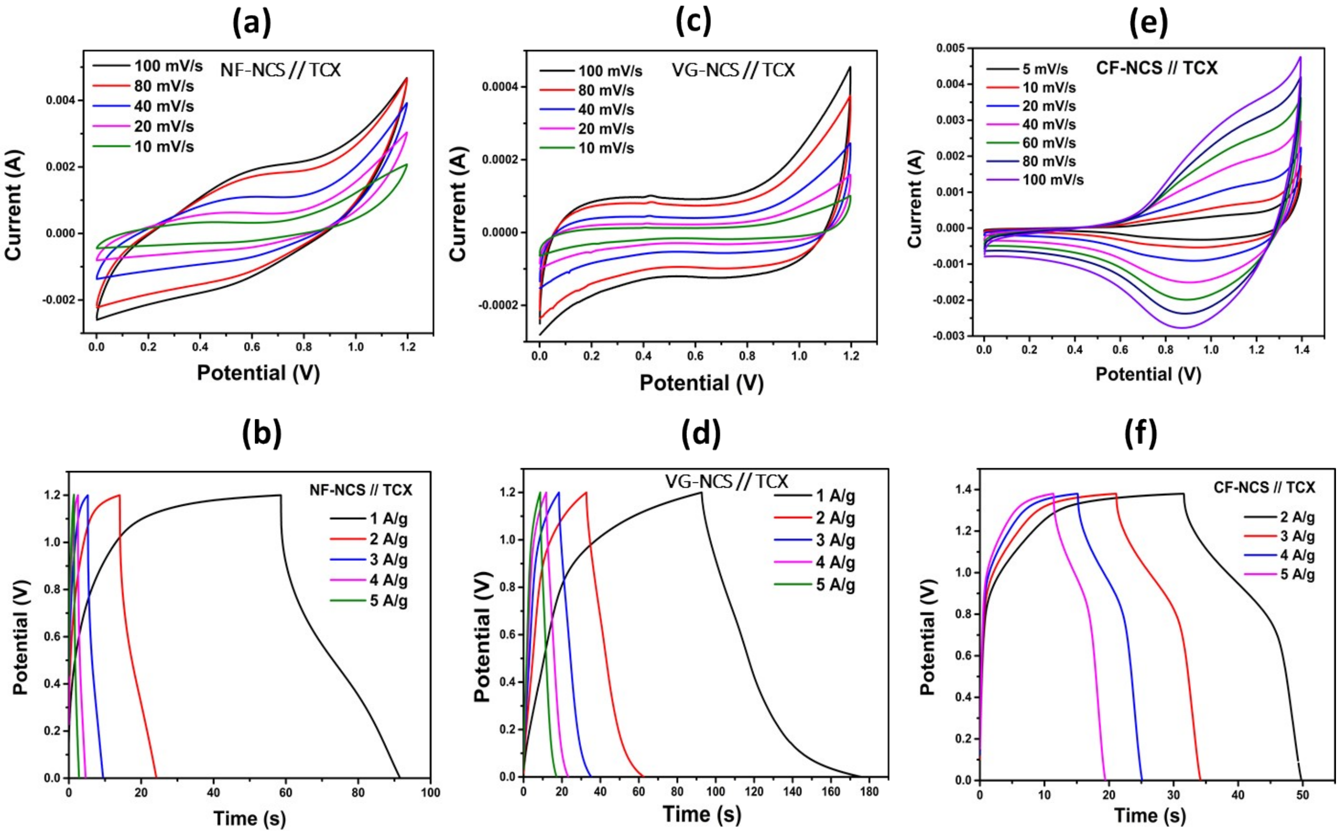

Fig-S5: (a-b) the CV and GCD curves of NF-NCS// $Ti_3C_2T_x$, (c-d) CV and GCD curve of VG-NCS// $Ti_3C_2T_x$; (e-f) CV and GCD curve of CF-NCS// $Ti_3C_2T_x$ Asymmetric assembly

Figure S6

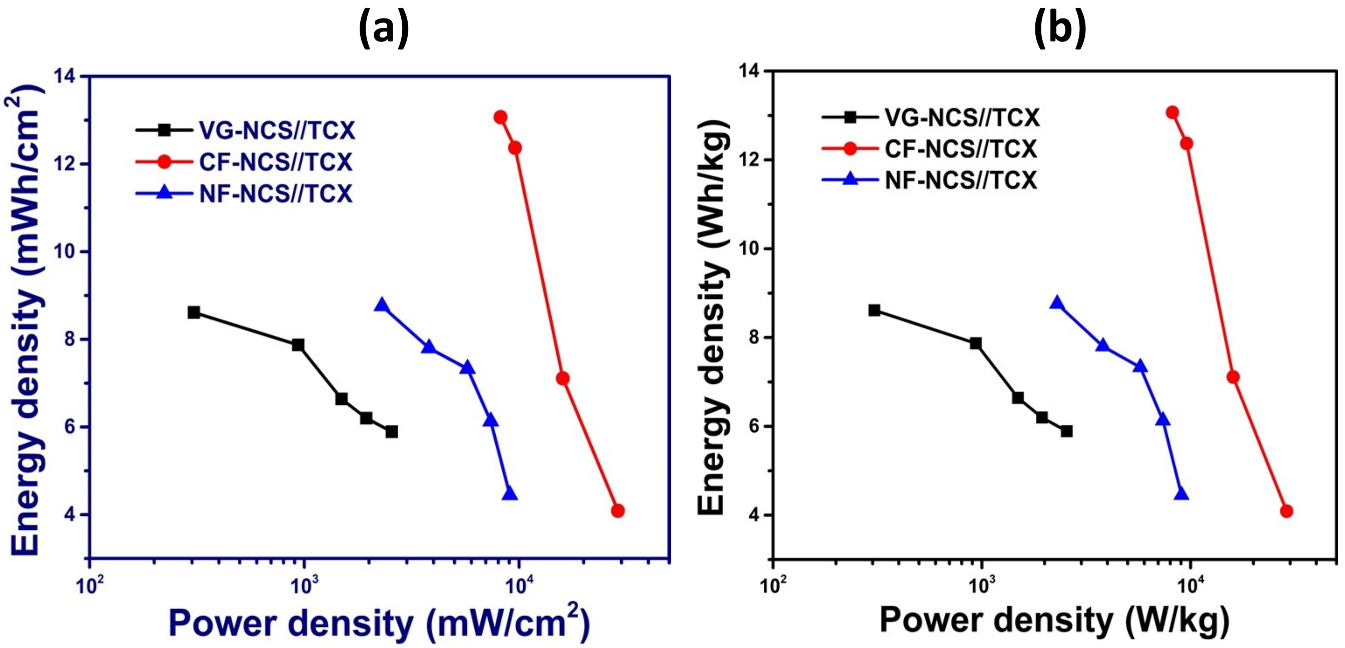

Fig-S6: (a) comparative Ragone plot- areal energy density (mWh/cm^2) vs. areal power density (mW/cm^2) and (b) gravimetric energy density (Wh/kg) vs. power density (W/kg) of all the supercapacitor electrodes of NCS grown on VG, CF and NF substrate

Table S1: Comparison of electrochemical performance of Nickel cobalt sulfides and its supercapacitor performance in symmetric and asymmetric assembly.

Material	Specific capacitance (F/g)	Areal capacitance (mF/cm^2)	Energy density (Wh/kg)	Power density (W/kg)	Areal Energy density (mWh/cm^2)	Areal Power density (mW/cm^2)	Cycling stability %/ cycles
CF-NCS // CF-NCS	167.28	-	11.38	8550	-	-	66/3000
NF-NCS // NF-NCS	116.62	-	4.5	5170	-	-	61/3000
VG-NCS // VG-NCS	64.26	-	1.5	2177	-	-	81/3000
CF-NCS // TCX	54.57	48.6	14.86	28870	13.22	25412	79/5000
NF-NCS // TCX	49	45	8.76	9051	8.74	8050	77/5000
VG-NCS // TCX	47	42.83	8.61	2555	8.57	2290	85/5000
